# Molecular differences in Alzheimer's disease between male and female patients determined by integrative network analysis

**DOI:** 10.1111/jcmm.13852

**Published:** 2018-11-05

**Authors:** Lin‐Lin Sun, Song‐Lin Yang, Hui Sun, Wei‐Da Li, Shu‐Rong Duan

**Affiliations:** ^1^ Department of Neurology The First Affiliated Hospital of Harbin Medical University Harbin China; ^2^ Department of Critical Care Medicine The First Affiliated Hospital of Harbin Medical University Harbin China; ^3^ Pharmaceutical Experiment Teaching Center College of Pharmacy Harbin Medical University Harbin China

**Keywords:** Alzheimer's disease, integrative network, sex differences, temporal and spatial patterns

## Abstract

Alzheimer's disease (AD) is a complex neurodegenerative disease and the most common cause of dementia among the elderly. There has been increasing recognition of sex differences in AD prevalence, clinical manifestation, disease course and prognosis. However, there have been few studies on the molecular mechanism underlying these differences. To address this issue, we carried out global gene expression and integrative network analyses based on expression profiles (GSE84422) across 17 cortical regions of 125 individuals with AD. There were few genes that were differentially expressed across the 17 regions between the two sexes, with only four (encoding glutamate metabotropic receptor 2, oestrogen‐related receptor beta, kinesin family member 26B, and aspartoacylase) that were differentially expressed in three regions. A pan‐cortical brain region co‐expression network analysis identified pathways and genes (eg, glycogen synthase kinase 3β) that were significantly associated with clinical characteristics of AD (such as neurofibrillary score) in males only. Similarity analyses between region‐specific networks indicated that male patients exhibited greater variability, especially in the superior parietal lobule, dorsolateral prefrontal cortex and occipital visual cortex. A network module analysis revealed an association between clinical traits and crosstalk of sex‐specific modules. An examination of temporal and spatial patterns of sex differences in AD showed that molecular networks were more conserved in females than in males in different cortical regions and at different AD stages. These findings provide insight into critical molecular pathways governing sex differences in AD pathology.

## 1 INTRODUCTION

Alzheimer's disease (AD) is a complex neurodegenerative disorder that is the most common cause of dementia among the elderly.^1^, ^2^ It is characterized by accumulation of β‐amyloid plaques and neurofibrillary tangles in areas of the brain associated with cognition such as the cortex and hippocampus.^3^ Advanced age is the strongest predictor of AD; however, there is increasing evidence for sex differences in its prevalence, clinical manifestation, disease course and prognosis.^4^, ^5^, ^6^ Previous studies have shown that male and female AD patients also exhibit differences in cognitive performance and behavior.^4^ Sexual dimorphism in brain structure and function also contributes to sex differences in AD.^5^, ^7^

Genetic studies on the association between single‐nucleotide polymorphisms and AD have suggested that the risk of AD is greater in female than in male carriers of the *apolipoprotein* (*Apo*)*E4* allele.^5^, ^8^, ^9^, ^10^ An interaction effect of *ApoE4* polymorphisms and sex on tacrine response has also been reported.^11^ Disruption of some metabolic processes can lead to cell death, neuronal loss and progressive decline from mild cognitive impairment to AD dementia.^6^ neuropathology and cerebral glucose metabolism vary by age and sex and may be important factors in AD aetiology,^12^, ^13^ while insulin metabolism also differs between the two sexes.^14^ For instance, oestrogen has protective effects in the brain, and its loss during menopause may lead to deficits in brain metabolism and consequently, to AD.^15^, ^16^ Although many studies have investigated the molecular and genetic basis of AD, there is little information on the sex differences because of the difficulty in constructing a global map based on traditional biological and clinical approaches within the limited scale of these studies.

These limitations can be overcome to some extent by high‐throughput experimental and bioinformatics technologies. For example, a data set of gene expression across 19 cortical regions was obtained from 1053 postmortem brain samples of 125 male and female individuals.^2^ In this study, we investigated the molecular basis of sex differences in AD across 19 cortical regions using a network construction approach combined with genome‐wide transcriptome data. The analysis had four components: (a) identification of differentially expressed genes (DEGs); (b) mapping of gene co‐expression networks; (c) examination of crosstalk in sex‐specific modules relevant to AD pathology; and (d) evaluation of dynamic networks to determine sex differences in AD progression. Our findings provide novel insight into sex differences in AD that can be applied to the development of and more personalized and effective strategies for disease diagnosis and treatment.

## 2 MATERIALS AND METHODS

### 2.1 Data sources

We downloaded raw AD‐related mRNA expression profiles (GSE84422) from the GEO database, and the data were derived from three different Affymetrix platforms (Human Genome U133A Array, Human Genome U133B Array, and Human Genome U133 Plus 2.0 Array). This gene expression dataset reflected changes in gene expression associated with multiple clinical and neuropathological traits in 1053 postmortem brain samples across 19 brain regions from 125 individuals with varying severity of dementia and AD neuropathology. All data were normalized using the MAS5 algorithm. For each expression profile, probe sets were mapped to Entrez Gene IDs. If multiple probe sets corresponded to the same gene, then their expression values were averaged. Meanwhile, probe sets corresponding to multiple genes were removed. Genes appearing in all of the expression profiles were included in the analysis. Additionally, given the absence of samples of two brain regions (amygdala and nucleus accumbens), the remaining 17 brain regions were used in the analysis. Microarray data set information according to experiment and brain region is listed in Table S1.

### 2.2 Construction of a co‐expression network

Gene co‐expression network analysis was performed to identify gene modules with coordinated expression patterns. Based on samples from male and female patients (probable AD and definite AD), we calculated PCC between all pairs of genes after separately normalizing microarray data (|PCC| > 0.9 and *P* < 0.01). Using the male and female networks, we generated three additional networks, including male‐ and female‐specific networks and an overlap network.

### 2.3 Identification of network modules

Network modules comprise a subset of interconnected genes in the gene co‐expression network and can provide more detailed information about AD pathogenesis. We identified network modules across two co‐expression networks using the MCODE algorithm with default settings, which is a plug‐in of Cytoscape software (http://www.cytoscape.org/).

### 2.4 Pathway enrichment analysis

DAVID 6.7 was used to carry out functional enrichment analysis. GO BP terms and KEGG pathways with a *P* value < 0.01 were selected as statistically significant terms.

### 2.5 K‐M analysis

A Kaplan‐Meier (K‐M) analysis was used to evaluate the correlation between gene expression and AD clinical characteristics. Like the survival analysis, which compares two patients groups in terms of the fraction of patients who live for a certain amount of time, it was used here to evaluate the significance of differences in clinical characteristics of AD between the two groups of samples, which is divided by the expression level of a gene of interest. The log‐rank test was used to evaluate the significance of differences in survival time.

### 2.6 Definition of dynamic score

To evaluate the dynamic nature of AD, we defined a dynamic score for different brain regions separately in male and female patients as follows:

$$\text{Score}=1-\frac{\text{overlapNum}}{\text{normalNum}}$$

where overlapNum represents overlapping co‐expressed gene pairs in all samples (normal, possible AD, probable AD and definite AD) and normalNum represents co‐expressed gene pairs in normal samples.

## 3 RESULTS

### 3.1 Identification of DEGs

**Figure 1:**
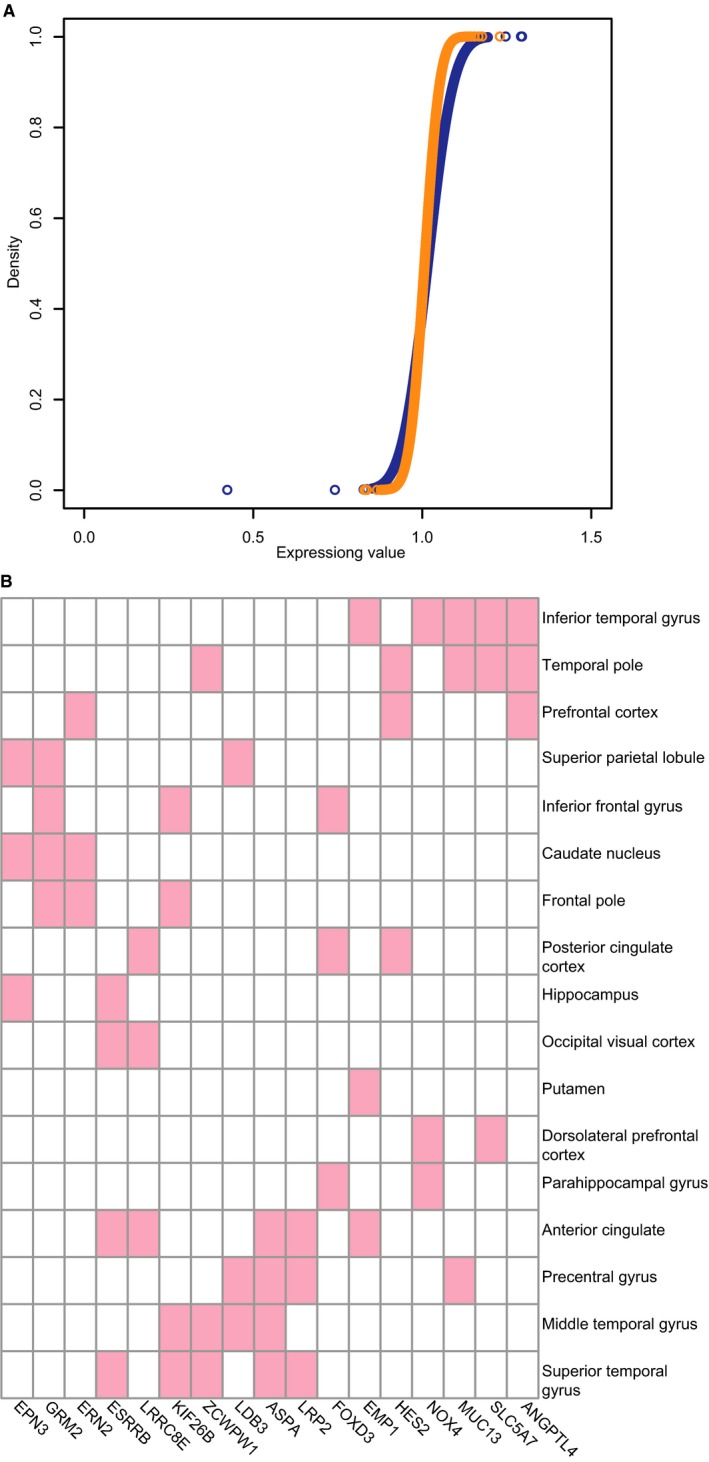
A, Distribution of gene expression values in male (blue) and female (orange) samples. B, differentially expressed genes (DEGs) in 17 cortical regions

To identify genes that are differentially expressed between male and female AD patients, we downloaded raw AD‐related mRNA expression profiles (GSE84422) from the Gene Expression Omnibus (GEO) database (Table S1). The samples in this dataset represented four neuropathological categories: normal, possible AD, probable AD and definite AD. DEGs between normal and probable and definite AD samples were identified. After normalization and analysis, we did not find any genes that were differentially expressed between normal subjects and male and female AD patients (fold difference >1.5 or <2/3 and *P* < 0.05; Table S2 and Figure 1A). We next examined DEGs across 17 cortical regions and found that the number varied greatly (ranging from 7 to 46) across different regions (Tables 1 and S2). Most of these were region specific, and only a small fraction were shared by multiple regions. For example, there were 17 DEGs in over three regions and only four (encoding glutamate metabotropic receptor [GRM]2, oestrogen‐related receptor beta [ESRRB], kinesin family member 26B, and aspartoacylase [ASPA]) shared by four regions (Figure 1B). GRM2 belongs to the family of G protein‐coupled receptors that inhibit the cyclic AMP cascade.^17^, ^18^ Proneurogenic GRM2 antagonists were shown to improve learning and reduce anxiety in a mouse model of AD.^19^ As such, GRM2 and GRM3 agonists are being developed for the treatment of psychotic symptoms associated with AD.^20^ ASPA encodes an enzyme that catalyses the conversion of *N*‐acetyl‐l‐aspartic acid (NAA) to aspartate and acetate.^21^ NAA is abundant in the brain, and its hydrolysis by ASPA is thought to contribute to the maintenance of white matter.^22^ NAA levels were found to be abnormal during early AD in a mouse model, which was associated with deficits in mitochondrial oxidative phosphorylation.^23^ ESRRB is a protein with similarity to the receptor of oestrogen, which regulates key processes in AD pathogenesis and underlies sex differences in AD.^24^, ^25^ Moreover, brain oestrogen deficiency has been linked to increased risk of developing AD in females.^24^

**Table 1:** Differentially expressed genes (DEGs) in different cortical regions

Brain region	Gene symbol
Anterior cingulate	ASPA BCL2L1 CDKN2B MEGF6 EMP1 ESRRB GBP1 GBP2 IFIT2 LRP2 ATP8B1 PLD1 TNFRSF10D RASSF9 C1RL TTC12 PECR PIEZO2 TRPM8 LRRC8E ZNF814
Caudate nucleus	DNAH9 ELAVL4 GLRB GRIA4 GRM2 KCNC1 KCNJ6 PTPN3 ERN2 NTNG1 ZFR2 EHD3 EPN3 PCDHB13 CA10 RANBP17 GRTP1 TRIM5 LOC643733
Dorsolateral prefrontal cortex	ADH1B EPHA1 GABRA1 KCNC3 TBXAS1 ZNF3 GDF15 SLC25A15 LAMB4 USP24 NME7 NOX4 RNFT1 SCARA3 TTC22 MAGOHB TRPC7 SLC5A7 GALNT12
Frontal pole	GRM2 HOXA3 MUSK ERN2 PARPBP KIF26B WDR60
Hippocampus	ZNHIT2 EPHA1 ESRRB FABP1 ITPR3 OSM PENK THBD HAP1 AURKB MTHFD2 FCF1 FBXO40 SLC6A20 EPN3
Inferior frontal gyrus	RUNX3 GRM2 PSD4 DKFZP434L187 FOXD3 FOXP3 ZNF771 KIF26B
Inferior temporal gyrus	RHOH CD44 CP CST6 EMP1 GBP2 GSTT1 LTBR NPHP1 PAX9 PDE9A SCNN1G SLAMF1 SLC6A2 TBXAS1 TP73 YBX3 ADAM7 SOCS3 SLC13A2 MAP3K6 ADAMTS1 STXBP5L FST MTHFD2 LAMB4 PSD4 PTPN22 FBXO24 PKD2L2 GPR132 ADGRE2 NOX4 ZBTB7B ANGPTL4 TOR4A TTC12 SPATA7 MUC13 TRPC7 SLC5A7 MICALL2 EFCC1 MOGAT2 UNC93B1 ESPN
Middle temporal gyrus	ASPA CD37 ERBB3 MOBP ZNF536 LDB3 ICOSLG RASGRP3 PLEKHG3 ZCWPW1 KIF26B AGPAT4 EFCC1 CFAP43 CFAP70
Occipital visual cortex	ESRRB EVC LRP5 TRPM1 CAPN15 TBX1 MZB1 SCARA3 FAM118A FGD6 TEX13A TMEM185B VASH2 LRRC8E
Parahippocampal gyrus	ANGPT2 APOC3 CD5 CFTR F2RL2 HSPA1L LAMA3 NRAP PAX3 PDE9A TACR1 S1PR2 KCNK7 ADAM28 FOXD3 PKD2L2 ANGPTL3 OBP2A NOX4 IMPG2 IL23A NUP62CL ESRP1 KDM4D MYH7B EBF2 HAND2‐AS1 MOGAT2 HKDC1 RNF32
Posterior cingulate cortex	ANPEP BCL6 CD44 MEGF6 FANCA KRT19 MMP11 YBX3 TNFRSF10D SLC13A2 MTL5 PTPN22 FOXD3 NDOR1 HES2 DCHS2 DPPA4 CASC5 DDX31 OTUB2 LRRC8E SPC24
Precentral gyrus	ASPA LRP2 MMP15 NFKBIB PIP4K2A PLD1 SLC14A1 GPRC5A MTL5 CEP104 LDB3 ICOSLG RASGRP3 PLEKHG3 SPATA6L SPATA7 MUC13 MTUS1 CLPB
Prefrontal cortex	ARG1 BTC CYP19A1 DBH DSC3 GHSR HNF4G KCNJ6 TPH1 ZNF214 KIF14 NR1H4 AGR2 ERN2 SLC22A7 ADAM30 ADAMTS6 IKZF3 ATP11B PRND ANGPTL3 ANGPTL4 GMIP GINS2 MYO3A HES2 CNGB3 GIPC2 FAM46C GALNT10 ZNF286A ZNF250 OTUB2 KDM8 DHX57 TRMT44 RAD21L1
Putamen	EGR1 EMP1 GCNT2 TNC ROR1 REG3A UBE3C GRAP GPR157 MCM3AP‐AS1 ARAP1
Superior parietal lobule	ACVRL1 ELAVL4 GRM2 PIP4K2A SLC12A4 XIST ZFY USP9Y PCSK7 AURKB DPP3 PLA2G16 LDB3 DRICH1 GPR173 EPN3 ATP8B2 MYH7B FA2H MCTP1 BAIAP2L2 NETO2 RAD21L1 ZNF814
Superior temporal gyrus	ASPA C7 ERBB3 ESRRB HK2 IGFBP1 LRP2 MYBL1 SIX1 SLC19A1 IKZF2 SMC5 TCL6 CDH19 SETD4 ZCWPW1 KIF26B P3H2 FAM46A PCDHB3 LOC100506282
Temporal pole	CD36 CD37 CFTR ELF3 EYA4 GHSR HAL IGHG1 NKTR SLC5A1 PCSK7 GDF15 LAMC3 ZNF234 PRDM5 LPAR3 TNFAIP8 ANGPTL4 HES2 TOR4A ZCWPW1 ARMC4 MUC13 ZNF286A NGB PRODH2 SLC5A7 ERAP2 RANBP17 NSUN7 CPED1 C16orf95

We investigated whether specific gene ontology (GO) terms were enriched among the DEGs observed in 17 brain regions using Database for Annotation, Visualization and Integrated Discovery (DAVID). The number of biological process (BP) terms (level 3) varied markedly among brain regions (*P* < 0.05; Table S3). Most GO terms were region specific; only seven were shared by two regions. Interestingly, six of the seven terms were shared by inferior temporal gyrus and posterior cingulate cortex (Table S2), while only four DEGs were shared by these two regions, indicating that although there were sex differences in 17 cortical regions, molecular similarities still existed.

### 3.2 Gene co‐expression network analysis

**Figure 2:**
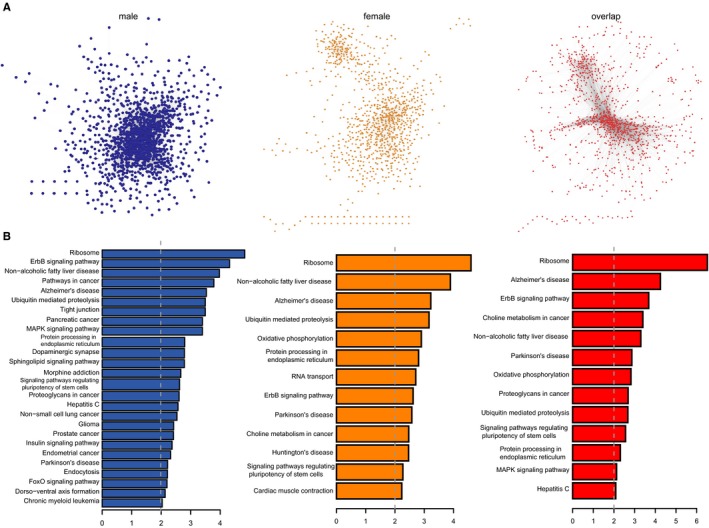
Co‐expression network analysis. A, From left to right: male‐ and female‐specific networks and overlap network. B, Significantly enriched pathways for the networks in (A)

Alzheimer's disease is a complex disease involving multiple interacting signalling pathways. We carried out a co‐expression network analysis to examine differences in the interactions of co‐expressed genes between males and females for each of the 17 different brain regions. For males, the overall network contained 80 739 edges and 1483 genes whereas for females, the overall network contained 25 772 edges including 1227 genes, with 21 984 correlations (Jaccard coefficient = 0.26) shared by the two co‐expression networks. Based on this data, we generated an overlap network as well as male‐ and female‐specific networks (Figure 2A). To determine the functional categories of the three networks, we performed Kyoto Encyclopedia of Genes and Genomes (KEGG) pathway annotation using DAVID; the top pathways are listed in Figure 2B. Unexpectedly, although each network had a unique set of genes, they were also enriched in some common pathways. For example, ribosome (hsa03010) was the most significant pathway in both male‐ and female‐specific networks, followed by neurodegenerative disease‐related pathways such as Parkinson's disease (hsa05012) and ErbB signaling (hsa04012). The latter modulates hippocampal γ oscillations, which are important for higher brain processes and are associated with AD.^26^, ^27^ AD (hsa05010) was observed in both male‐ and female‐specific pathways (Figure 2B), although the annotated genes differed between the two sexes (Figure S1A,B). For example, in the AD pathway, glyceraldehyde 3‐phosphate dehydrogenase and glutamate ionotropic receptor *N*‐methyl‐d‐aspartate (NMDA) type subunit 2D were specific to female and male AD patients, respectively. The NMDA receptor is involved in long‐term potentiation, and an activity‐dependent increase in synaptic transmission efficiency is thought to underlie some types of learning and memory.^28^ Interestingly, glutamate‐mediated toxicity in hippocampal HT‐22 cells is enhanced by testosterone,^29^ which contributes to sex differences in AD.^7^

**Figure 3:**
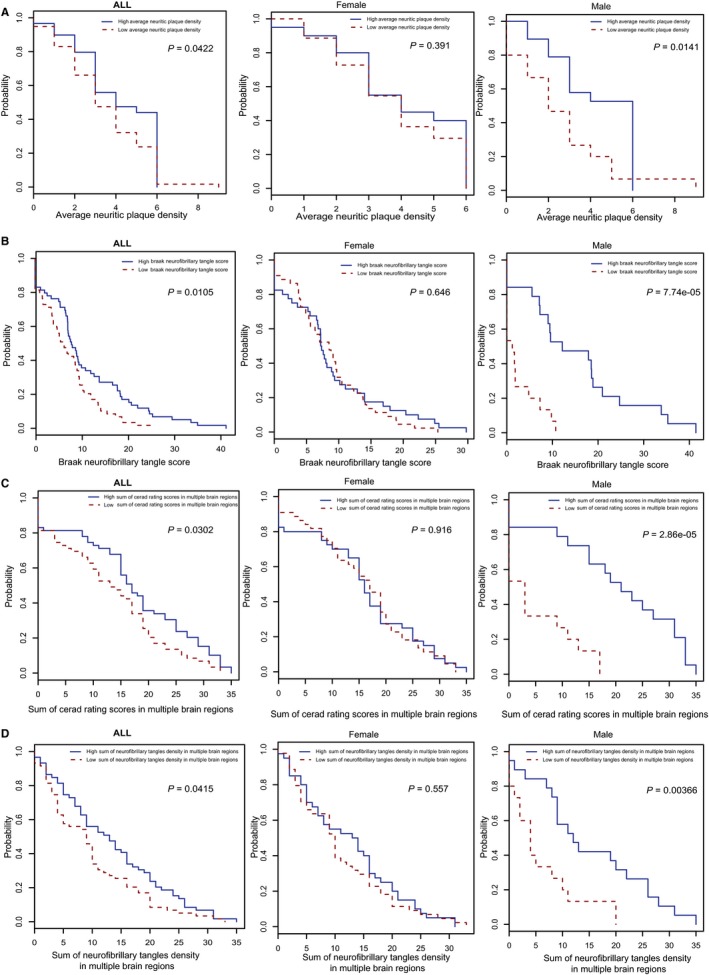
Kaplan‐Meier test of GSK3β based on (A) average neuritic plaque density; B, Braak neurofibrillary tangle score; C, sum of Consortium to Establish a Registry for Alzheimer's disease rating scores in multiple brain regions; and D, sum of neurofibrillary tangle density in multiple brain regions

Similar observations were made for the insulin signaling pathway (hsa04910) in males. Glycogen synthase kinase (GSK)3β (black circle in Figure S2) is related to neurofibrillary tangle formation,^30^, ^31^ and its phosphorylation is associated with cardiac adaptation in mice; moreover, sex differences in GSK3β expression have been linked to the survival of adherent leukemic progenitors.^32^, ^33^ We performed a K‐M analysis to determine whether AD patients with different clinical characteristics could be distinguished based on GSK3β expression level. The results showed that GSK3β expression was significantly related to four clinical traits in AD including “Braak neurofibrillary tangle score,” “Average neuritic plaque density,” “Sum of Consortium to Establish a Registry for Alzheimer's Disease rating scores in multiple brain regions” and “Sum of neurofibrillary tangle density in multiple brain regions” (Figure 3A‐D). Interestingly, GSK3β expression was more significant in males than in females, indicating that it could be a male‐specific molecular marker for AD.

**Figure 4:**
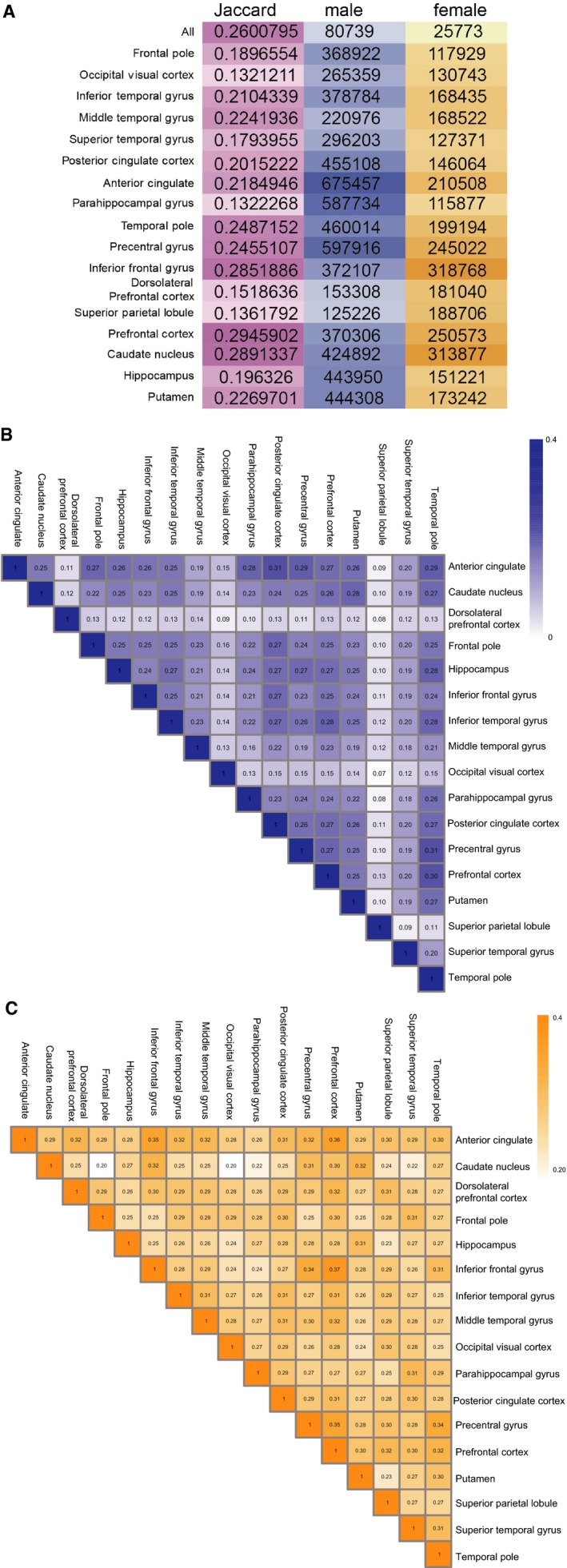
A, Numbers of co‐expressing network edges and Jaccard coefficients of 17 cortical regions between males and females. B,C, Jaccard coefficients across the 17 regions in males (B) and females (C)

We constructed 34 co‐expression networks for the 17 cortical regions in males and females and compared the network edges of the two sexes using the Jaccard index. Jaccard coefficients of the 17 cortical regions varied within a relatively small range from 0.13 (prefrontal cortex) to 0.29 (dorsolateral prefrontal cortex) (Figure 4A), indicating that males and females have cortical region‐specific molecular mechanisms but also some similarities. We further examined the conservation of modules across the 17 brain regions by computing the similarity between all pairs of modules based on network edges using the Jaccard index for males and females. We found that the range of Jaccard coefficients across the 17 regions was larger in males (0.07‐0.31) than in females (0.20‐0.37) (Figure 4B,C). Interestingly, three regions (superior parietal lobule, dorsolateral prefrontal cortex and occipital visual cortex) differed significantly from other regions. Among these, the superior parietal lobule had the smallest average Jaccard coefficient. Furthermore, these three regions showed greater differences between males and females (Figure 4A), suggesting that they contribute to the observed sex differences in AD.^34^, ^35^

### 3.3 Crosstalk between sex‐specific modules related to AD pathology

**Figure 5:**
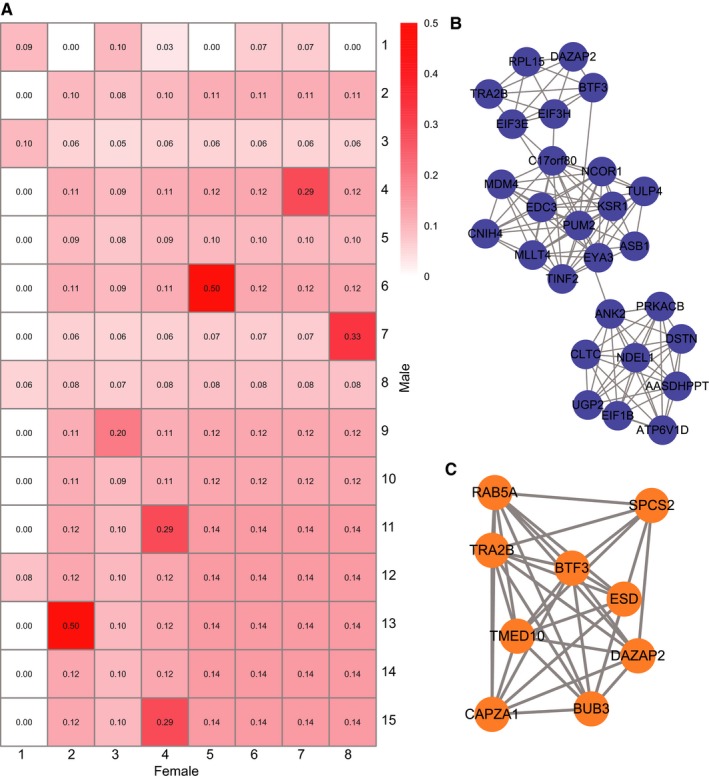
Crosstalk between sex‐specific modules. A, Jaccard coefficient between male and female modules. B, Male‐specific module. C, Female‐specific module

Alzheimer's disease is a genetically heterogeneous disease, in which various genes interact in a single biological module; these genes tend to show similar expression profiles and can encode components of a multi‐protein complex, pathway or single organelle.^36^ We used the MCODE algorithm to identify modules in male and female co‐expression networks and investigate their interactions (Pearson's correlation coefficient [PCC] > 0.95 and *P* < 0.01).^37^ There were 15 modules in the male co‐expression network (designated as M1‐M15) and eight in the female network (designated as F1‐F8) (Table S4). By calculating Jaccard coefficients between the male and female modules, we determined that most of the modules were sex specific but other were shared (Figure 5A). The highest Jaccard coefficients were between M13 and F2 and between M6 and F5 (Jaccard coefficients = 0.5; Figure 5A). The intersecting genes between M6 and F5 were Sortilin (SORT)1 and Krüppel‐like factor (KLF)13. Genetic variants of the former have been linked to increased risk of AD.^38^ We found here that the expression level of the latter was significantly associated with four clinical characteristics of AD in males (Figure S3).

Interestingly, although M1 and F1 were the largest modules, they were also sex specific, with the lowest average Jaccard coefficients (Figure 5A). The two modules are shown in Figure 5B,C. Functional annotation revealed that genes in male module 1 were involved in cellular component assembly (GO: 0022607) and translational initiation (GO: 0006413), while female module 1 was only enriched in establishment of protein localization (GO: 0045184).

### 3.4 Networks exhibiting sex differences in AD progression

**Figure 6:**
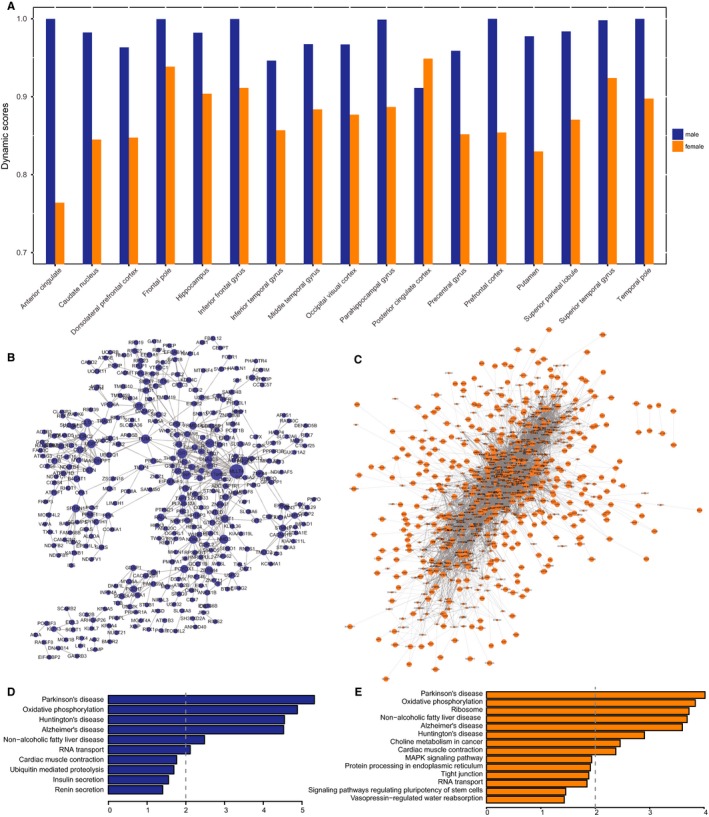
A, Dynamic scores in males and females. B,C, Conserved networks of males (B) and females (C) across 17 brain regions in definite AD. D,E, Significantly enriched pathways for conserved networks in males (D) and females (E)

Alzheimer's disease is a chronic progressive disease, and studies have shown that different brain regions vary in terms of vulnerability to AD. To investigate sex differences in AD development, we compared various brain regions in terms of molecular differences in males and females at different AD stages. We first constructed co‐expression networks of each of four stages (normal, possible AD, probable AD and definite AD) in each brain region in male and female samples, discarding regions with fewer than four samples. We then calculated the dynamic score using the number of edges of corresponding networks in each brain region. The scores ranged from 0 to 1, with a higher score indicating greater changes in AD at different disease stages. Notably, we found that the range of dynamic scores in 17 brain regions was lower in males than in females (0.91‐0.99 vs 0.76‐0.94; *P* = 0.03906), indicating that molecular alterations during AD progression were greater in males (Table 2 and Figure 6A).

**Table 2:** The number of edges and dynamic scores in 17 brain regions for males and females

	Normal	Possible AD	Probable AD	Definite AD	Overlap	Score
Frontal pole	552223 (777879)	86973 (433862)	12790 (270613)	432782 (167646)	261 (47810)	0.999527365 (0.938538)
Occipital visual cortex	527769 (351564)	261508 (543264)	N/A (233300)	155842 (254039)	17399 (43249)	0.967032925 (0.876981)
Inferior temporal gyrus	198612 (316910)	106157 (375176)	N/A (273127)	433929 (307081)	10671 (45335)	0.946272129 (0.856947)
Middle temporal gyrus	430642 (688423)	93704 (422968)	N/A (298747)	235468 (370383)	14030 (49419)	0.967420735 (0.8837)
Superior temporal gyrus	355013 (688423)	11511 (339404)	N/A (624409)	296203 (112770)	687 (52336)	0.99806486 (0.923977)
Posterior cingulate cortex	358495 (894835)	176717 (250078)	N/A (563781)	562718 (177025)	31813 (45752)	0.911259571 (0.948871)
Anterior cingulate	611561 (311564)	48430 (677160)	10426 (326324)	930858 (383965)	136 (73530)	0.999777618 (0.763997)
Parahippocampal gyrus	617064 (361783)	5231 (422968)	N/A (402422)	507297 (149879)	671 (40953)	0.998912593 (0.886802)
Temporal pole	565399 (680216)	41958 (308743)	8334 (391154)	548532 (282634)	87 (69685)	0.999846126 (0.897555)
Precentral gyrus	14236 (361783)	116408 (588083)	N/A (383589)	663736 (358904)	585 (12468)	0.958906996 (0.851795)
Inferior frontal gyrus	240356 (1260395)	205034 (399279)	6214 (817409)	563532 (325913)	85 (111796)	0.999646358 (0.911301)
Dorsolateral prefrontal cortex	888593 (478807)	371582 (611679)	N/A (419392)	153308 (290019)	32605 (73000)	0.963307161 (0.847538)
Superior parietal lobule	614800 (577592)	287773 (666463)	N/A (482516)	65717 (269236)	9949 (74781)	0.983817502 (0.87053)
Prefrontal cortex	416658 (775398)	40038 (999755)	6977 (554521)	543842 (293055)	40 (113178)	0.999903998 (0.854039)
Caudate nucleus	237057 (599185)	45562 (411294)	N/A (436888)	528826 (500654)	4177 (92857)	0.982379765 (0.845028)
Hippocampus	335324 (289056)	29830 (270554)	N/A (320649)	599797 (220469)	6002 (27797)	0.982100893 (0.903835)
Putamen	61558 (574247)	48685 (400815)	N/A (300052)	515028 (279224)	1383 (59840)	0.977533383 (0.829789)
Overlap	6 (1421)	0 (1794)	0 (729)	553 (5806)		

The numbers in the brackets represents female data.

We also investigated molecular differences in 17 brain regions in four AD stages (normal, possible AD, probable AD and definite AD). In male AD patients, there were almost no edges overlapping among the 17 brain regions except in definite AD (Table 2), indicating that there are some common pathways or interactions when the disease symptoms are sufficiently obvious to make a diagnosis. Similar observations were made in females: there were more overlapping edges in definite AD than in the other stages. Conserved networks in males and females across 17 brain regions in definite AD are shown in Figure 6B,C, respectively. We found that the network for females was much more dense than that of males. The average degree in the female network was 17 as compared to 3.2 in the male network. A KEGG pathway analysis showed that genes in male and female networks were enriched in pathways associated with AD (hsa05010) and other neurodegenerative disorders such as Parkinson's disease (Figure 6D,E). Oxidative phosphorylation (hsa00190) was a pathway common to the male and female networks, suggesting that this is a universal and coordinated molecular process in multiple brain regions in patients of both sexes. Indeed, decreased oxidative phosphorylation is a feature of AD.^39^ We also observed sex‐specific pathways such as choline metabolism in cancer (hsa05231) in females. This is consistent with reports that AD patient brains exhibit abnormal choline metabolism.^40^

## 4 DISCUSSION

This is the first large‐scale study characterizing sex differences in AD pathology based on transcriptome analysis of multiple brain regions. We found that the number of DEGs varied greatly across brain regions and that few genes were shared by different regions. For example, ESRRB (oestrogen‐related receptor beta) is differentially expressed in four regions. Oestrogens is found to be involved in cognition and memory and also contribute to gender‐related differences in AD.^41^ After menopause, the decline of oestrogen levels in the brain may make neurons more susceptible to age‐related neurodegenerative processes.^42^ Yue et al^24^ found that, compared with APP23 transgenic control mice, oestrogen‐deficient APP23 mice exhibited greatly reduced brain oestrogen and early onset and increased beta amyloid peptide (Abeta) deposition. Selective oestrogen receptor modulators have been potentially used for treatment of AD. Thus, ESRRB may be a novel potential therapeutic target for AD. We also found that most of GO terms were region specific. Surprisingly, there were no genes that were differentially expressed overall between normal and AD samples in males and females, suggesting that DEG analysis may not be sufficient to identify sex differences in AD.

Genes are organized into functional networks; gene co‐expression networks reflect transcriptional relationships between genes in various biological contexts, including disease states.^43^ In the present study, we carried out an integrative network analysis to identify critical pathways and genes responsible for sex differences at different stages of dementia. We constructed an overall gene co‐expression network along with networks of 17 different cortical regions for males and females and then generated male‐ and female‐specific networks and an overlap network. The functional annotation indicated that there were pathways common to the two sexes including AD (hsa05010) and insulin signalling (hsa04910) pathways, although the annotated genes differed. In the latter, we identified the GSK3β gene as being significantly associated with four clinical traits in male but not female AD patients, suggesting that it contributes to sex differences in AD. GSK3β was found be involve in tau hyperphosphorylation, which appears to have an important role in the formation of NFTs.^30^ Interestingly, oestrogens were shown to directly interact with GSK3β and lowered its activity, resulting in decreased hyperphosphorylation of tau.^44^ Additionally, we identified functional modules in males and females and calculated the Jaccard coefficient between these modules. We found that most of the modules were sex specific, although there was some overlap between modules. The highest Jaccard coefficient (0.5) was between M13 and F2 and between M6 and F5. Intersecting genes between M6 and F5 included SORT1 and KLF13; the latter was also significantly related to four clinical traits in male AD patients. These analyses demonstrate that the co‐expression modules captured meaningful gene–gene interaction relationships underlying sex differences in AD.

We investigated sex differences in AD progression and found that dynamic alterations in AD progression were greater in males than in females; however, the female network was denser than that of males, suggesting that temporal and spatial changes in AD are more dramatic in males. Functional annotation based on the KEGG pathway analysis revealed that genes in male and female networks were both enriched in AD (hsa05010) and neurodegenerative disease pathways.

In conclusion, the results of this study not only reveal sex‐specific networks and pathways associated with AD, but also provide novel insights into molecular mechanisms underlying sex differences in the clinical symptoms of AD. These findings can provide a basis for the development of more personalized strategies for AD diagnosis and treatment.

## CONFLICT OF INTEREST

The authors declare that there is no conflict of interests regarding the publication of this paper.
