# Supplementary material for: Molecular differences in Alzheimer's disease between male and female patients determined by integrative network analysis
Source: J Cell Mol Med. 2018 Nov 5;23(1):47–58. doi: 10.1111/jcmm.13852 (PMC6307813; doi:10.1111/jcmm.13852)
Supplement: Supplementary file 1 [file JCMM-23-47-s001.pdf]

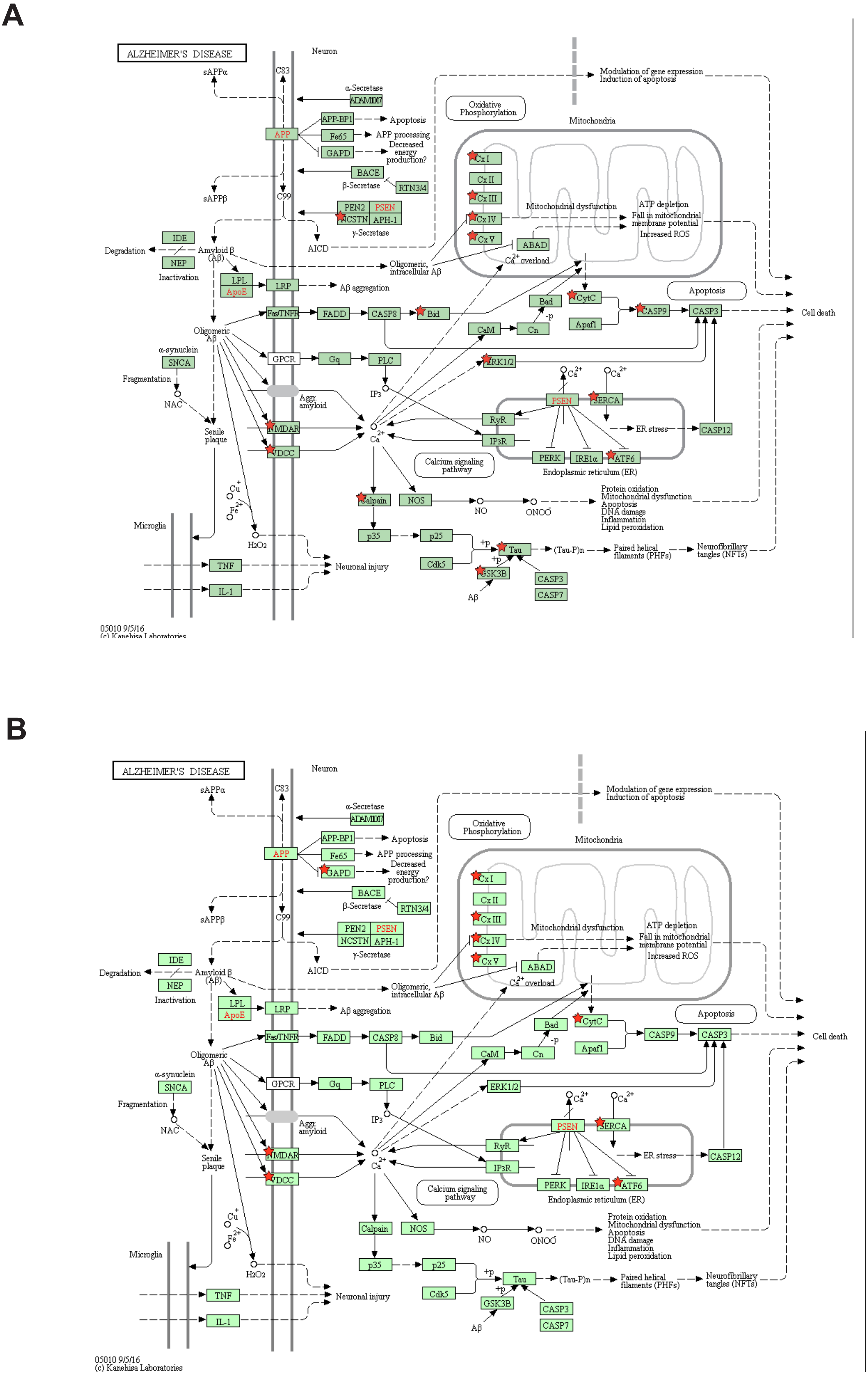
A
ALZHEIMER'S DISEASE
Neuron
sAPPα
C83
α-Secretase
ADAM10/17
APP-BP1
Apoptosis
APP
Fe65
APP processing
GAPD
Decreased energy production?
BACE
β-Secretase
RTN3/4
sAPPβ
C99
PEN2
PSEN
NCSTN
APH-1
γ-Secretase
AICD
Oxidative Phosphorylation
Modulation of gene expression
Induction of apoptosis
Mitochondria
Cx I
Cx II
Cx III
Cx IV
Cx V
ABAD
Mitochondrial dysfunction
ATP depletion
Fall in mitochondrial membrane potential
Increased ROS
Ca2+ overload
IDE
Degradation
Amyloid β (Aβ)
NEP
Inactivation
Oligomeric, intracellular Aβ
LPL
ApoE
LRP
Aβ aggregation
Fas/TNFR
FADD
CASP8
Bid
Bad
-p
CytC
Apaf1
CASP9
CASP3
Apoptosis
Cell death
CaM
Cn
Oligomeric Aβ
α-synuclein
SNCA
Fragmentation
NAC
Senile plaque
GPCR
Gq
PLC
IP3
ERK1/2
Aggr. amyloid
Ca2+
PSEN
SERCA
RyR
IP3R
ER stress
CASP12
NMDAR
VDCC
Calcium signaling pathway
PERK
IRE1α
ATF6
Endoplasmic reticulum (ER)
Cu+
Fe2+
Calpain
NOS
NO
ONOO-
Protein oxidation
Mitochondrial dysfunction
Apoptosis
DNA damage
Inflammation
Lipid peroxidation
Microglia
H2O2
p35
p25
Cdk5
+p
Tau
(Tau-P)n
Paired helical filaments (PHFs)
Neurofibrillary tangles (NFTs)
GSK3B
CASP3
CASP7
Aβ
TNF
IL-1
Neuronal injury
05010 9/5/16
(c) Kanehisa Laboratories
B
ALZHEIMER'S DISEASE
Neuron
sAPPα
C83
α-Secretase
ADAM10/17
APP-BP1
Apoptosis
APP
Fe65
APP processing
GAPD
Decreased energy production?
BACE
β-Secretase
RTN3/4
sAPPβ
C99
PEN2
PSEN
NCSTN
APH-1
γ-Secretase
AICD
Oxidative Phosphorylation
Modulation of gene expression
Induction of apoptosis
Mitochondria
Cx I
Cx II
Cx III
Cx IV
Cx V
ABAD
Mitochondrial dysfunction
ATP depletion
Fall in mitochondrial membrane potential
Increased ROS
Ca2+ overload
IDE
Degradation
Amyloid β (Aβ)
NEP
Inactivation
Oligomeric, intracellular Aβ
LPL
ApoE
LRP
Aβ aggregation
Fas/TNFR
FADD
CASP8
Bid
Bad
-p
CytC
Apaf1
CASP9
CASP3
Apoptosis
Cell death
CaM
Cn
Oligomeric Aβ
α-synuclein
SNCA
Fragmentation
NAC
Senile plaque
GPCR
Gq
PLC
IP3
ERK1/2
Aggr. amyloid
Ca2+
PSEN
SERCA
RyR
IP3R
ER stress
CASP12
NMDAR
VDCC
Calcium signaling pathway
PERK
IRE1α
ATF6
Endoplasmic reticulum (ER)
Cu+
Fe2+
Calpain
NOS
NO
ONOO-
Protein oxidation
Mitochondrial dysfunction
Apoptosis
DNA damage
Inflammation
Lipid peroxidation
Microglia
H2O2
p35
p25
Cdk5
+p
Tau
(Tau-P)n
Paired helical filaments (PHFs)
Neurofibrillary tangles (NFTs)
GSK3B
CASP3
CASP7
Aβ
TNF
IL-1
Neuronal injury
05010 9/5/16
(c) Kanehisa Laboratories
