# Supplementary figures and images for: Molecular differences in Alzheimer's disease between male and female patients determined by integrative network analysis
Source: J Cell Mol Med. 2018 Nov 5;23(1):47–58. doi: 10.1111/jcmm.13852 (PMC6307813; doi:10.1111/jcmm.13852)

# INSULIN SIGNALING PATHWAY

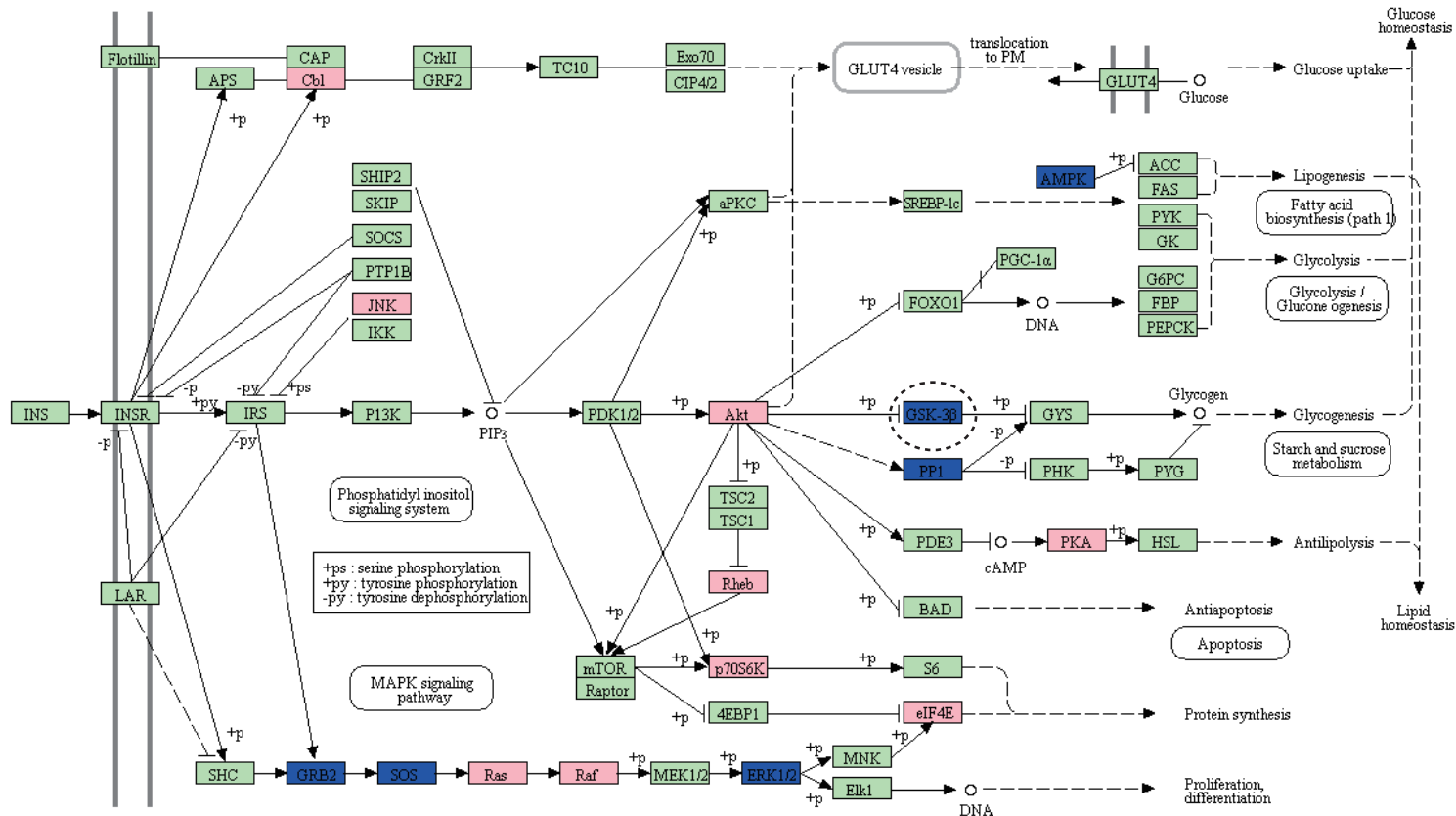

Supplement: Supplementary file 2 [file JCMM-23-47-s002.pdf]

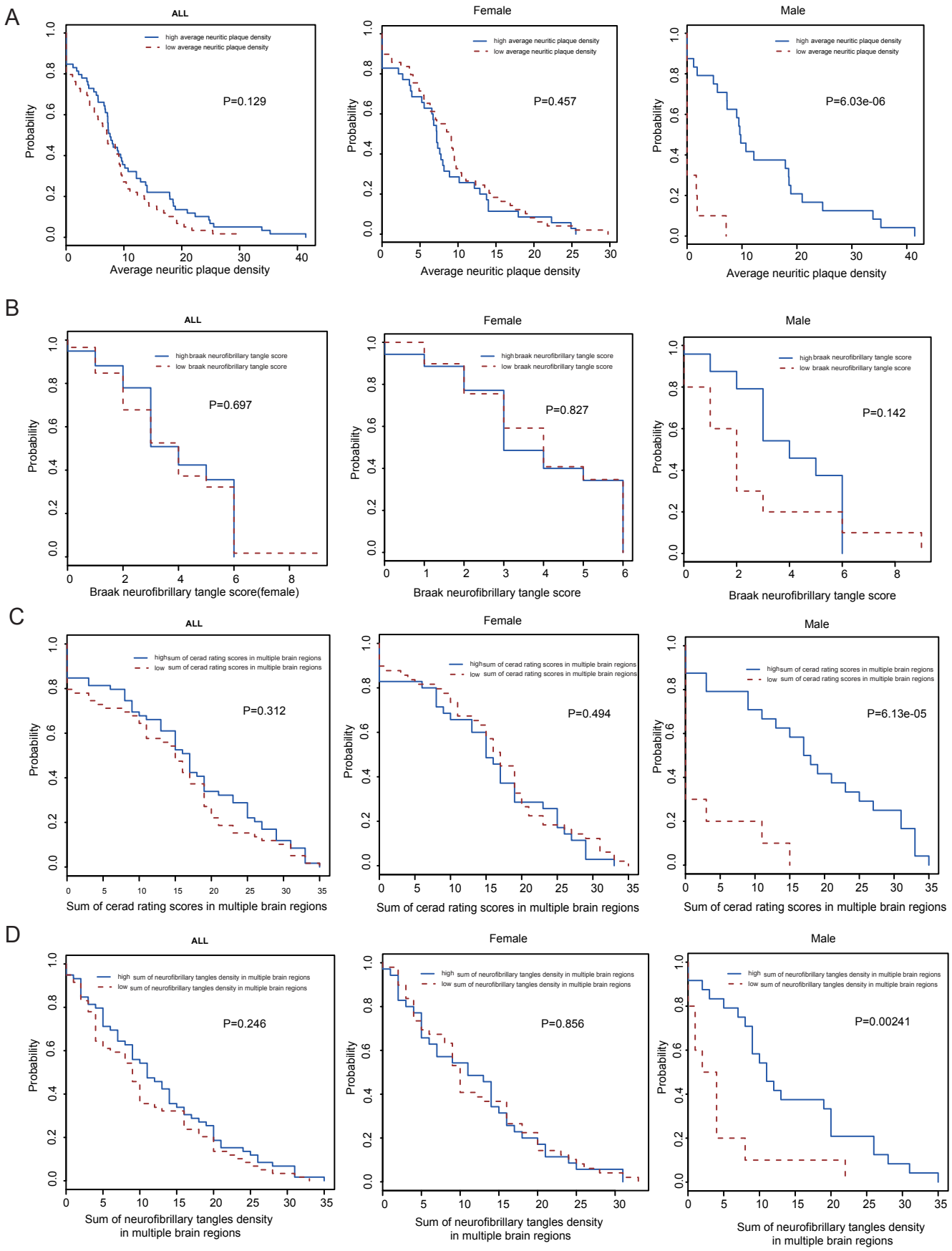

Supplement: Supplementary file 3 [file JCMM-23-47-s003.pdf]
