# Supplementary material for: Molecular differences in Alzheimer's disease between male and female patients determined by integrative network analysis
Source: J Cell Mol Med. 2018 Nov 5;23(1):47–58. doi: 10.1111/jcmm.13852 (PMC6307813; doi:10.1111/jcmm.13852)
Supplement: Supplementary file 4 [file JCMM-23-47-s004.docx]

**Table S1.** Numbers of samples in different brain region

| The brain region | male | | | | female | | | |
| --- | --- | --- | --- | --- | --- | --- | --- | --- |
|  | Normal | Possible AD | Probable AD | definite AD | Normal | Possible AD | Probable AD | definite AD |
| Frontal Pole | 14 | 8 | 4 | 18 | 16 | 18 | 18 | 30 |
| Occipital Visual Cortex | 12 | 10 | 2 | 8 | 14 | 18 | 22 | 20 |
| Inferior Temporal Gyrus | 12 | 8 | 2 | 14 | 16 | 20 | 20 | 24 |
| Middle Temporal Gyrus | 12 | 8 | 2 | 18 | 16 | 18 | 20 | 22 |
| Superior Temporal Gyrus | 10 | 4 | 0 | 12 | 18 | 26 | 18 | 32 |
| Posterior Cingulate Cortex | 10 | 10 | 2 | 16 | 14 | 20 | 14 | 30 |
| Anterior Cingulate | 12 | 6 | 4 | 14 | 20 | 16 | 20 | 26 |
| Parahippocampal Gyrus | 12 | 4 | 2 | 12 | 18 | 22 | 16 | 34 |
| Temporal Pole | 12 | 6 | 4 | 16 | 16 | 24 | 18 | 20 |
| Precentral Gyrus | 4 | 8 | 2 | 14 | 6 | 18 | 22 | 24 |
| Inferior Frontal Gyrus | 8 | 8 | 4 | 12 | 14 | 22 | 12 | 26 |
| Dorsolateral Prefrontal Cortex | 14 | 10 | 0 | 10 | 18 | 16 | 22 | 24 |
| Superior Parietal Lobule | 12 | 10 | 2 | 8 | 14 | 20 | 16 | 18 |
| Prefrontal Cortex | 10 | 6 | 4 | 14 | 12 | 18 | 20 | 28 |
| Caudate Nucleus | 8 | 6 | 2 | 14 | 14 | 18 | 20 | 22 |
| Hippocampus | 12 | 6 | 2 | 14 | 10 | 22 | 22 | 22 |
| Putamen | 6 | 6 | 2 | 14 | 12 | 18 | 20 | 26 |
| Amygdala | 9 | 0 | 0 | 0 | 6 | 7 | 12 | 17 |
| Nucleus Accumbens | 7 | 0 | 0 | 0 | 6 | 10 | 11 | 17 |
