# Supplementary material for: Molecular differences in Alzheimer's disease between male and female patients determined by integrative network analysis
Source: J Cell Mol Med. 2018 Nov 5;23(1):47–58. doi: 10.1111/jcmm.13852 (PMC6307813; doi:10.1111/jcmm.13852)
Supplement: Supplementary file 5 [file JCMM-23-47-s005.docx]

Table S2 Number of DEGs in different cortical regions

| cortical regions | Male/normal | Female/normal | female/male |
| --- | --- | --- | --- |
| All | 0 | 0 | 0 |
| Frontal Pole | 2 | 5 | 7 |
| Occipital Visual Cortex | 10 | 2 | 14 |
| Inferior Temporal Gyrus | 3 | 0 | 46 |
| Middle Temporal Gyrus | 5 | 0 | 15 |
| Superior Temporal Gyrus | 4 | 0 | 21 |
| Posterior Cingulate Cortex | 0 | 3 | 22 |
| Anterior Cingulate | 4 | 2 | 21 |
| Parahippocampal Gyrus | 2 | 2 | 30 |
| Temporal Pole | 3 | 1 | 32 |
| Precentral Gyrus | 22 | 36 | 19 |
| Inferior Frontal Gyrus | 3 | 3 | 8 |
| Dorsolateral Prefrontal Cortex | 6 | 1 | 19 |
| Superior Parietal Lobule | 4 | 1 | 24 |
| Prefrontal Cortex | 0 | 5 | 37 |
| Caudate Nucleus | 6 | 4 | 19 |
| Hippocampus | 2 | 7 | 15 |
| Putamen | 15 | 0 | 11 |
| Amygdala | No sample | 23 | No sample |
| Nucleus Accumbens | No sample | 42 | No sample |
