# Supplementary material for: Molecular differences in Alzheimer's disease between male and female patients determined by integrative network analysis
Source: J Cell Mol Med. 2018 Nov 5;23(1):47–58. doi: 10.1111/jcmm.13852 (PMC6307813; doi:10.1111/jcmm.13852)
Supplement: Supplementary file 6 [file JCMM-23-47-s006.docx]

Table s3 GO terms in different cortical regions

| Anterior Cingulate | GO:0032534~regulation of microvillus assembly |
| --- | --- |
|  | GO:0032528~microvillus organization |
|  | GO:0030031~cell projection assembly |
|  | GO:0007093~mitotic cell cycle checkpoint |
|  | GO:0060491~regulation of cell projection assembly |
| Caudate Nucleus | GO:0006811~ion transport |
|  | GO:0034220~ion transmembrane transport |
|  | GO:0034765~regulation of ion transmembrane transport |
|  | GO:0055085~transmembrane transport |
|  | GO:0034762~regulation of transmembrane transport |
|  | GO:0043269~regulation of ion transport |
|  | GO:0099536~synaptic signaling |
|  | GO:0044765~single-organism transport |
|  | GO:0007399~nervous system development |
| Hippocampus | GO:0033043~regulation of organelle organization |
| Inferior Frontal Gyrus | GO:0045934~negative regulation of nucleobase-containing compound metabolic process |
|  | GO:0031327~negative regulation of cellular biosynthetic process |
|  | GO:0009890~negative regulation of biosynthetic process |
|  | GO:0051172~negative regulation of nitrogen compound metabolic process |
|  | GO:0019219~regulation of nucleobase-containing compound metabolic process |
|  | GO:0031324~negative regulation of cellular metabolic process |
|  | GO:0031326~regulation of cellular biosynthetic process |
|  | GO:0034654~nucleobase-containing compound biosynthetic process |
|  | GO:0018130~heterocycle biosynthetic process |
|  | GO:0019438~aromatic compound biosynthetic process |
| Inferior Temporal Gyrus | GO:0010648~negative regulation of cell communication |
|  | GO:1902531~regulation of intracellular signal transduction |
|  | GO:0000165~MAPK cascade |
|  | GO:0023014~signal transduction by protein phosphorylation |
|  | GO:0070302~regulation of stress-activated protein kinase signaling cascade |
|  | GO:0031098~stress-activated protein kinase signaling cascade |
|  | GO:0009968~negative regulation of signal transduction |
|  | GO:1902532~negative regulation of intracellular signal transduction |
|  | GO:0048513~animal organ development |
|  | GO:0030097~hemopoiesis |
|  | GO:0080135~regulation of cellular response to stress |
|  | GO:0048534~hematopoietic or lymphoid organ development |
|  | GO:0050709~negative regulation of protein secretion |
|  | GO:0048871~multicellular organismal homeostasis |
|  | GO:0002520~immune system development |
|  | GO:0030098~lymphocyte differentiation |
|  | GO:0009966~regulation of signal transduction |
|  | GO:0048731~system development |
|  | GO:1903531~negative regulation of secretion by cell |
|  | GO:0045321~leukocyte activation |
|  | GO:0042110~T cell activation |
|  | GO:0051048~negative regulation of secretion |
|  | GO:0050714~positive regulation of protein secretion |
|  | GO:0051224~negative regulation of protein transport |
|  | GO:0001894~tissue homeostasis |
|  | GO:0007159~leukocyte cell-cell adhesion |
| Occipital Visual Cortex | GO:0090596~sensory organ morphogenesis |
|  | GO:0001525~angiogenesis |
|  | GO:0002053~positive regulation of mesenchymal cell proliferation |
|  | GO:0048514~blood vessel morphogenesis |
|  | GO:0010464~regulation of mesenchymal cell proliferation |
|  | GO:0007423~sensory organ development |
|  | GO:0001944~vasculature development |
|  | GO:0008284~positive regulation of cell proliferation |
| Parahippocampal Gyrus | GO:0048878~chemical homeostasis |
|  | GO:0035561~regulation of chromatin binding |
|  | GO:0006796~phosphate-containing compound metabolic process |
|  | GO:0006082~organic acid metabolic process |
|  | GO:0050994~regulation of lipid catabolic process |
| Posterior Cingulate Cortex | GO:1903037~regulation of leukocyte cell-cell adhesion |
|  | GO:0022407~regulation of cell-cell adhesion |
|  | GO:1902532~negative regulation of intracellular signal transduction |
|  | GO:0007159~leukocyte cell-cell adhesion |
|  | GO:0080135~regulation of cellular response to stress |
|  | GO:0016337~single organismal cell-cell adhesion |
|  | GO:0030098~lymphocyte differentiation |
|  | GO:0051249~regulation of lymphocyte activation |
|  | GO:0009968~negative regulation of signal transduction |
|  | GO:0002694~regulation of leukocyte activation |
|  | GO:0042110~T cell activation |
| Prefrontal Cortex | GO:0050728~negative regulation of inflammatory response |
|  | GO:0031348~negative regulation of defense response |
|  | GO:0032692~negative regulation of interleukin-1 production |
|  | GO:0012501~programmed cell death |
| Putamen | GO:0008284~positive regulation of cell proliferation |
|  | GO:0007267~cell-cell signaling |
|  | GO:0007166~cell surface receptor signaling pathway |
|  | GO:0070848~response to growth factor |
| Superior Temporal Gyrus | GO:0021545~cranial nerve development |
| Temporal Pole | GO:0022600~digestive system process |
|  | GO:0050892~intestinal absorption |
|  | GO:1901565~organonitrogen compound catabolic process |
