# Supplementary material for: Molecular differences in Alzheimer's disease between male and female patients determined by integrative network analysis
Source: J Cell Mol Med. 2018 Nov 5;23(1):47–58. doi: 10.1111/jcmm.13852 (PMC6307813; doi:10.1111/jcmm.13852)
Supplement: Supplementary file 7 [file JCMM-23-47-s007.docx]

Table S4 Modules in males and females

| Male | | | | |
| --- | --- | --- | --- | --- |
| Cluster | Score (Density*#Nodes) | Nodes | Edges | Node IDs |
| 1 | 8.692 | 27 | 113 | ANK2, EIF3H, NDEL1, ATP6V1D, TRA2B, EIF3E, EIF1B, DAZAP2, UGP2, RPL15, PUM2, EYA3, PRKACB, AASDHPPT, DSTN, TINF2, MDM4, MLLT4, EDC3, KSR1, NCOR1, ASB1, C17orf80, CNIH4, TULP4, BTF3, CLTC |
| 2 | 5 | 5 | 10 | SPIN1, TMOD2, GDAP1, KLHL12, DNM1L |
| 3 | 4.167 | 13 | 25 | IPO8, DNAJC3, NONO, ZNF84, LMBRD1, TAPT1, RALA, PPIA, EIF4ENIF1, CPSF7, BUB3, VPS26A, SPCS2 |
| 4 | 4 | 4 | 6 | STX6, RPL7, RNF34, SEPT7 |
| 5 | 4 | 6 | 10 | ANKS1B, ZNF667, CDKL2, RPS6KB1, ZBTB44, USP22 |
| 6 | 4 | 4 | 6 | EDIL3, SORT1, NIPAL3, KLF13 |
| 7 | 3.8 | 11 | 19 | UBFD1, EIF4E2, CACNA1B, NRXN3, MAP2, GPALPP1, AP4S1, HABP4, REPS1, NDUFAF5, RAB40C |
| 8 | 3.714 | 8 | 13 | RHOT1, KLHDC10, ZKSCAN8, BTBD7, MORF4L2, HERC1, SP1, RAB5A |
| 9 | 3.333 | 4 | 5 | PREPL, PRKAR1A, SYNJ1, KLC1 |
| 10 | 3.333 | 4 | 5 | COX6A1, UBE2Q1, PPME1, SPTAN1 |
| 11 | 3 | 3 | 3 | ATP5F1, UQCRC2, COPS4 |
| 12 | 3 | 3 | 3 | HSPD1, TMED10, RBM39 |
| 13 | 3 | 3 | 3 | RPLP1, RPS23, RPS27 |
| 14 | 3 | 3 | 3 | EFCAB14, CRTC3, DESI2 |
| 15 | 3 | 3 | 3 | PSMB7, ITGB1BP1, NDUFB4 |
| Female | | | | |
| 1 | 8.25 | 9 | 33 | BUB3, ESD, TRA2B, SPCS2, DAZAP2, TMED10, CAPZA1, BTF3, RAB5A |
| 2 | 4 | 4 | 6 | RPLP1, HUWE1, EEF1A1, RPS27 |
| 3 | 3.6 | 6 | 9 | PRKACB, ANK2, HIGD1A, PRKAR1A, AASDHPPT, RAB1A |
| 4 | 3.333 | 4 | 5 | ATP5F1, PCNP, NDUFB4, CLTC |
| 5 | 3 | 3 | 3 | SORT1, SCARB2, KLF13 |
| 6 | 3 | 3 | 3 | C17orf80, TINF2, CYP2U1 |
| 7 | 3 | 3 | 3 | RPL7, EIF3E, EIF3H |
| 8 | 3 | 3 | 3 | UBFD1, EIF4E2, NDUFAF5 |
